# Inefficient Building Electrification Will Require Massive Buildout of Renewable Energy and Seasonal Energy Storage

**DOI:** 10.1038/s41598-022-15628-2

**Published:** 2022-07-13

**Authors:** Jonathan J. Buonocore, Parichehr Salimifard, Zeyneb Magavi, Joseph G. Allen

**Affiliations:** 1 Center for Climate, Health, and the Global Environment, Harvard T.H. Chan School of Public Health, Boston, MA USA; 2 Present Address: College of Civil and Construction Engineering, Oregon State University, Corvallis, OR USA; 3 Department of Environmental Health, Harvard T.H. Chan School of Public Health, Boston, MA USA; 4 HEET, Cambridge, MA USA; 5 Present Address: Department of Environmental Health, Boston University School of Public Health, Boston, MA USA

**Keywords:** Climate-change mitigation, Climate-change policy, Energy policy, Energy efficiency, Environmental impact

## Abstract

Building electrification is essential to many full-economy decarbonization pathways. However, current decarbonization modeling in the United States (U.S.) does not incorporate seasonal fluctuations in building energy demand, seasonal fluctuations in electricity demand of electrified buildings, or the ramifications of this extra demand for electricity generation. Here, we examine historical energy data in the U.S. to evaluate current seasonal fluctuation in total energy demand and management of seasonal fluctuations. We then model additional electricity demand under different building electrification scenarios and the necessary increases in wind or solar PV to meet this demand. We found that U.S. monthly average total building energy consumption varies by a factor of 1.6×—lowest in May and highest in January. This is largely managed by fossil fuel systems with long-term storage capability. All of our building electrification scenarios resulted in substantial increases in winter electrical demand, enough to switch the grid from summer to winter peaking. Meeting this peak with renewables would require a 28× increase in January wind generation, or a 303× increase in January solar, with excess generation in other months. Highly efficient building electrification can shrink this winter peak—requiring 4.5× more generation from wind and 36× more from solar.

## Introduction

To date, most full-economy decarbonization pathways have heavily relied on electrification of energy use in buildings, transportation, and other sectors^1–3^. Along with climate benefits, electrification and consequent reduction in combustion energy sources would also have public health benefits by averting air pollution emissions^4^. Existing full-economy decarbonization models for the United States (U.S.) generally use yearly resolution—they do not incorporate monthly to seasonal variation in full energy demand, such as winter demand for heat^1–3,5–8^. Successful electrification of building heating will require the replacement of the absolute heating energy, along with the ability to manage seasonal fluctuation in demand, both of which are currently provided by the existing energy system to provide building heating.

In the U.S., 12% of residential buildings and 9.5% of commercial buildings use propane, oil, and/or wood, which can be stored on site or at distribution facilities, as a primary heating fuel^9,10^. Natural gas is a primary heating fuel for 42% of commercial buildings and 49% of residential buildings^9,10^. Natural gas also has a fleet of 388 active underground gas storage (UGS) facilities around the U.S., along with liquefied natural gas (LNG) facilities as part of its transmission and distribution system^11,12^. These facilities provide seasonal storage capacity for natural gas^11,12^. Successfully electrifying buildings, without relying on combustion fuels, requires replacing the energy supplied by these combustion sources along with their existing storage capability. This will increase the amount of electricity demand from buildings, which will need to be met by renewables in order to avoid reliance on combustion fuels^13^. Given the differences in seasonality between solar energy production and building heating energy demand, deployment of long-term energy storage may be key in enabling this demand to be met by renewables^3,8,13^.

Previous research on building electrification, decarbonization, and energy modeling have made a lot of progress in developing and evaluating different decarbonization pathways. However, this previous research has not evaluated (1) the degree of seasonal fluctuation in building energy demand, (2) how this seasonal fluctuation is currently managed, (3) how building energy demand and the degree of building energy demand may change under different building electrification scenarios, (4) what different building electrification scenarios may mean for grid demand, and (5) what it would take to meet this new demand using existing renewable energy technologies. To provide insight into potential paths forward for electrification of building heating, we fill these gaps by (1) evaluating the seasonal patterns in consumption of energy used for building heating and examining the degree of seasonal fluctuation, relying on monthly energy consumption, production, and gas flow data from the U.S. Energy Information Administration (EIA) from January 1973 through February 2020^14^, (2) focusing on natural gas and evaluating the role of UGS facilities in managing the asynchronicity between consumption of natural gas by buildings, and natural gas production^14^, (3) developing illustrative scenarios for how electricity demand could change as buildings are electrified by using coefficients of performance (COPs) from the literature^15–18^, (4) modeling how these different building electrification scenarios would affect the grid, and (5) calculating how much additional generation from wind and solar would be required to meet this demand using renewable electricity using present-day generation profiles for U.S. wind and solar.

## Methods

We obtained monthly energy data from the United States Energy Information Administration (EIA) from January 1973 (when monthly reporting starts) through February 2020 (the last month before energy disruptions due to COVID-19 and lockdowns in the U.S.)^11^. This dataset included monthly total energy consumption in residential and commercial buildings, monthly gas production and consumption data across all sectors, and monthly electricity generation and consumption across all sectors. We calculated monthly average energy consumption across both building types, and determined the seasonal fluctuations based on minimum and maximum monthly average energy consumption across the year. Similarly, we collated the natural gas production, consumption, and UGS activity data^11^, and determined the difference between the monthly average minimum and maximum to determine the size of the seasonal fluctuations. We tested the importance of the role of UGS in managing seasonal energy demand by comparing the r^2^ values from the two following regression models:

1. Total Gas Consumption ~ Natural Gas (Dry) Production
2. Total Gas Consumption ~ Natural Gas (Dry) Production + Natural Gas Storage Activity, Net

To build the prototypical electrification scenarios, we truncated the residential and commercial building energy consumption to the last decade (March 2010–February 2020) and aggregated the monthly averages to represent a recent seasonal profile of total energy consumption by buildings. We then split primary energy consumed by buildings into useful energy and losses using prototypical annual fuel use efficiency (AFUE) values of 95% for natural gas, 98% for electricity, 85% for coal, biomass, and other fossil fuels, and 100% for direct on-site use of geothermal and other renewables^15,16^. For electricity, we split the losses into fuel conversion losses, and then combine the 7% losses from transmission and distribution and the 5% losses from direct power plant use into one category. We then constructed a series of building electrification scenarios representing (1) 50% replacement of on-site fossil energy with electricity using technologies with COP of 1 (approximately the COP of baseboard resistance heating)^15^; (2) 100% replacement of in-building fossil energy with electricity using technologies with COP of 1^15^; (3) 100% replacement of in-building fossil energy with electricity using technologies with COP of 2 (approximately the COP of ASHPs)^15,17^; (4) 100% replacement of in-building fossil energy with electricity using technologies with COP of 4 (approximately the COP of GSHPs)^15,18^; and (5) 100% replacement of in-building fossil energy with electricity using technologies with COP of 6 (approximately the COP of networked GSHPs)^19^. We then calculated total primary energy demand and total electricity demand under each of these scenarios.

From these scenarios, we then calculated the change in total electricity demand, based on electricity consumption and production in the last decade (March 2010–February 2020). From monthly electricity generation patterns during that decade, we then calculated how much generation of wind and solar would have to increase to meet electricity demand under each scenario, and the maximum monthly electricity over-generation under each scenario^11^.

## Results

### The falcon curve: current seasonal fluctuations in building total energy use

**Figure 1:**
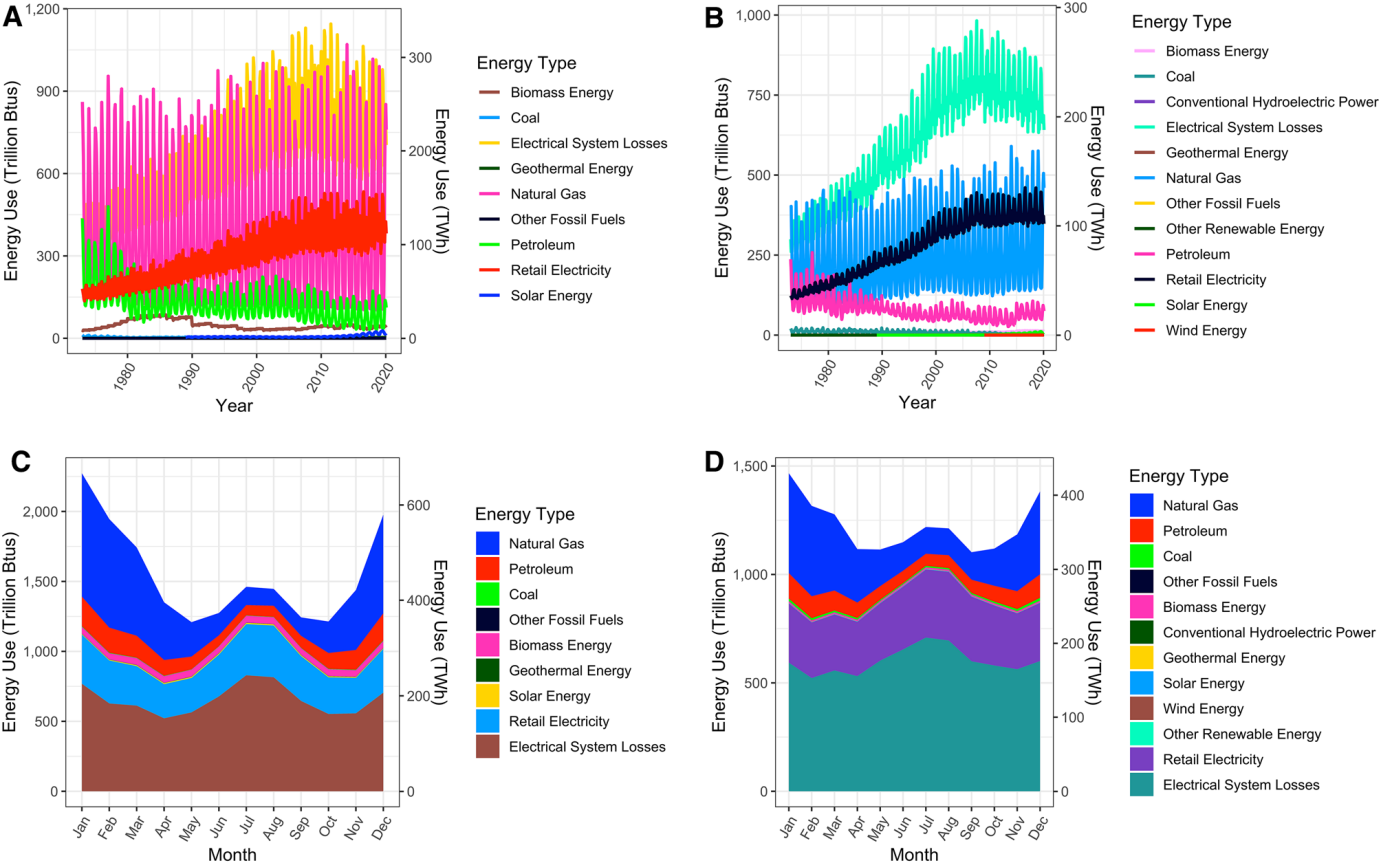
Energy consumption in buildings from January 1973 to February 2020. (**A**) Residential buildings; (**B**) commercial buildings; (**C**) monthly average in residential buildings; and (**D**) monthly average in commercial buildings.

Energy use in residential and commercial buildings have changed substantially over the last 50 years (Fig. 1). Electricity use and accompanying losses have increased from 1973 to 2010, and plateaued or decreased slightly since 2010; use of natural gas in commercial buildings has gone up slightly, and stayed roughly the same in residential buildings (Fig. 1A,B). All energy types have substantial seasonal variability in consumption, with a monthly profile resembling a falcon (Fig. 1C,D)—Peak total energy consumption occurring in December and January (heating season), a secondary peak in July and August (cooling season), and lowest in the transitional months April, May, September, and October. Monthly average total energy usage is lowest in May for residential buildings at 1205 trillion Btus (TBtus), and lowest in September for commercial at 1102 TBtus. Usage is highest in January, at 2270 TBtus for residential and 1466 for commercial. Gas responds to 77% of this increase in demand—increasing by 761 TBtus for residential buildings from August to January, and 338 TBtus for commercial buildings from January to July (Fig. 1C,D).

### The role of gas and underground gas storage facilities in managing seasonal fluctuations in heating energy demand

**Figure 2:**
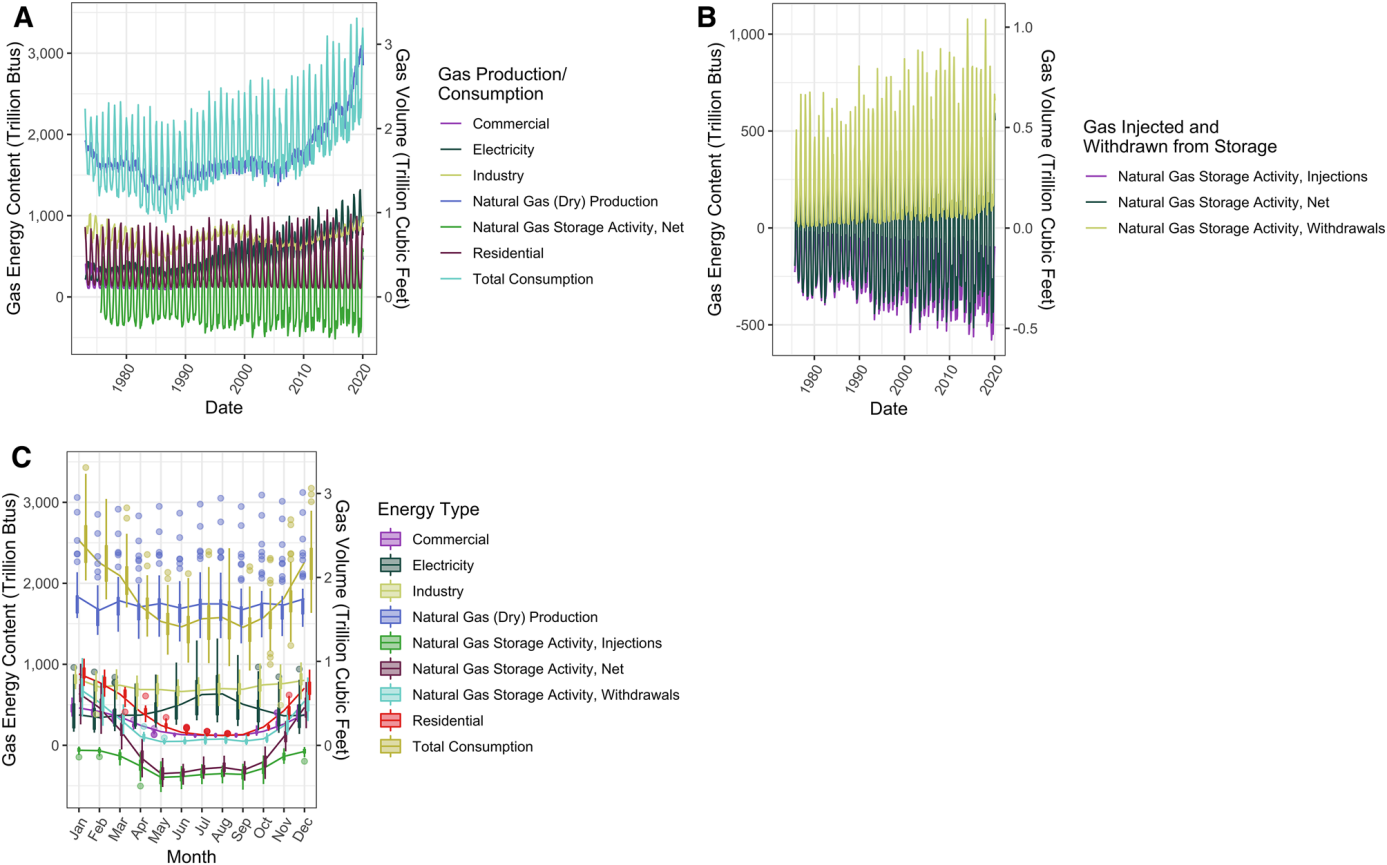
U.S. natural gas production, consumption, and storage from January 1973 to February 2020. (**A**) Monthly gas production and consumption; (**B**) monthly gas storage activity; and (**C**) monthly average gas flows.

Gas production and consumption across all sectors has stayed roughly the same from 1973 to around 2005, and increased after 2005, largely due to the growth of shale gas (Fig. 2A). Since 1973, monthly average total consumption of gas peaks at 2530 TBtus in January, and is lowest in September, at 1456 TBtus, with average seasonal fluctuation of 1074 TBtus (Figs. 2B,C). This seasonal variation is largely driven by consumption in buildings, with a secondary peak in July and August driven by electricity demand (Fig. 2B,C). However, gas production is fairly flat throughout the year, along with consumption in other sectors (Fig. 2B,C). This asynchronicity between gas production and consumption is largely managed by a network of 412 UGS facilities, 388 of which were active in 2019^20^. Around 14% of all gas produced in the U.S. annually is injected into UGS facilities for storage during the warmer months (April to October) and withdrawn from storage during the cooler months (November to March)^14,20^. During the average November-March heating season, 2341 TBtus is withdrawn from UGS facilities in total—21% of total gas consumption during those months (Fig. 2C). UGS has a strong role in balancing production and consumption of gas (regression *r*^2^ = 0.91 with UGS, and *r*^2^ = 0.37 without UGS). UGS is equivalent to a battery with 686 TWh of heat storage capacity, and peak discharge rate of 277 GW of heat. For comparison, at the end of 2018 in the U.S., the total power capacity of the U.S. grid-scale electric battery fleet was 869 MW, with a total electric storage capacity of 1236 MWh^21^. This does not include additional backup capacity, as the UGS fleet tends to keep reserves—monthly average stored working gas peaks in October at 3395 TBtus, and is lowest in March at 1529 TBtus.

### The “falcon curve” under different electrification scenarios

**Figure 3:**
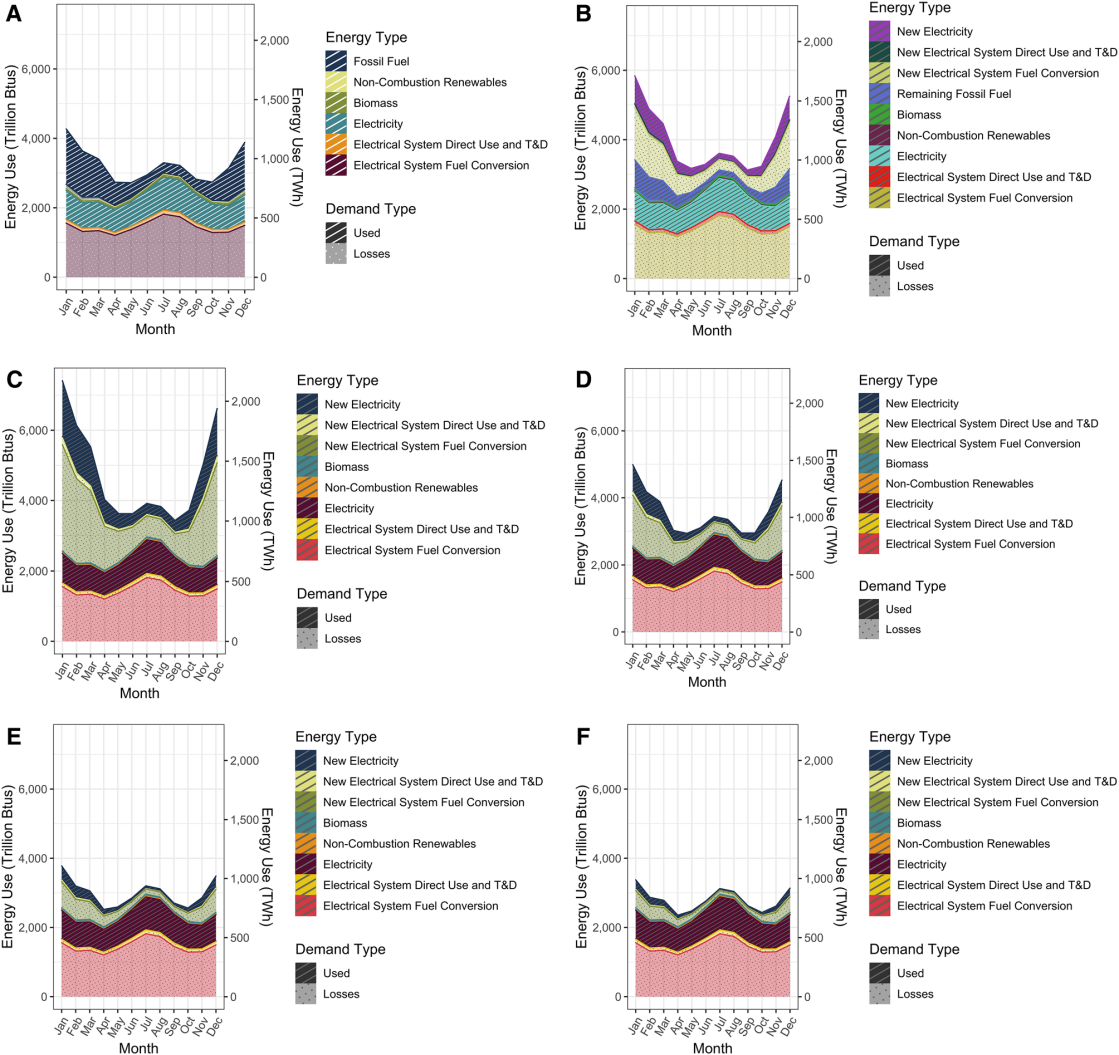
The “Falcon Curve”—Monthly average building total energy consumption from March 2010 to February 2020, and changes to building energy demand under different scenarios of building electrification with the current electrical grid. (**A**) current—all buildings’ energy demand. B-E are scenarios representing electrification of fossil energy use at (**B**) 50% conversion using technologies with a coefficient of performance (COP) of 1; (**C**) 100% conversion using technologies with a COP of 1; (**D**) 100% conversion using technologies with a COP of 2; (**E**) 100% conversion using technologies with a COP of 4; and (**F**) 100% conversion using technologies with a COP of 6.

Building energy demand fluctuates monthly, with a peak in winter that is a mixture of electricity and on-site fossil fuel use, a secondary summer peak that is largely electricity, and is lowest in the spring and fall months (Fig. 3A). The shape of the falcon curve varies under different hypothetical scenarios of building electrification (Fig. 3A–F). From March of 2010 through February of 2020, current monthly average total primary energy demand from buildings peaks in January, at 4271 TBtus, and is lowest in May, at 2722 TBtus (Fig. 3A)—a 1549 TBtus seasonal fluctuation. Electricity demand peaks in the summer 2883 TBtus in July (including ~ 66% losses from power plant losses and direct use, along with transmission losses), has a secondary peak at 2496 TBtus in January, and is lowest in April at 1943 TBtus (Fig. 3A), making a seasonal fluctuation of 940 TBtus. If 50% of current fossil building heating demand is met with technologies with a COP of 1^17^, total seasonal fluctuation in total energy demand would expand to 2715 TBtus from September to January. The additional demand on the electrical grid from electrifying heating would be enough to shift building demand from a summer peak to a winter peak, with 4917 TBtus in January, 3360 TBtus in July, and 2857 TBtus in May (Fig. 3B). If 100% of current fossil building energy is converted, the fluctuation in total energy demand expands to 3980 TBtus—3430 TBtus in September to 7410 TBtus in January (Fig. 3C). The expanse of this gap decreases as the COP for space heating technology increases (Fig. 3B–F). With a COP of 6^19,22^, the seasonal fluctuation in total energy demand decreases to 1022 TBtus—a peak of 3375 TBtus in January, 3122 TBtus in July, and 2353 TBtus in April.

### Managing the falcon curve on the electrical grid

**Figure 4:**
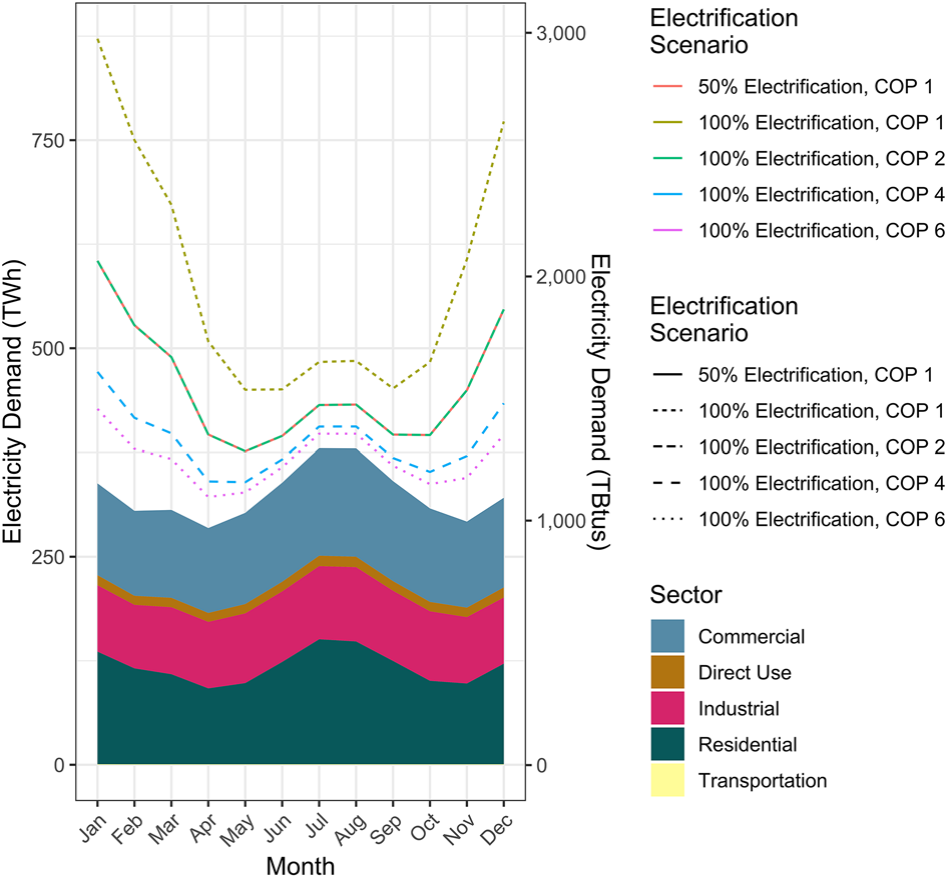
Current monthly total electricity demand by sector from March 2010 to February 2020, and projected changes to total building energy demand under different building electrification scenarios using technology with varying COPs. Solid area represents current demand, different electrification scenarios are represented using both color and line style.

Even under our most efficient scenario, using technologies with a COP of 6, electrifying building heating will put substantial additional demand on the electrical grid (Fig. 4), effectively superimposing the falcon curve onto the electrical grid. Currently, January electrical demand is 338 TWh. Under full building electrification with technologies with a COP of 1, total January demand increases by 534 TWh, to 872 TWh, surpassing the summer peak (Fig. 4). With technologies with a COP of 6, total demand in January increases by 89 TWh (~ 21%) to 427 TWh, higher than the summer peak (Fig. 4). Even under the most efficient prototypical COP, building electrification presents a fundamental shift in electrical grid seasonal dynamics, from a summer peak to a winter peak.

**Figure 5:**
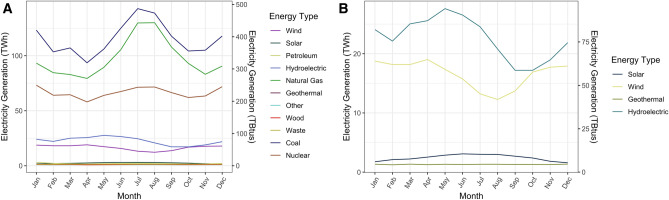
Monthly average electricity generation by source from March 2010 to February 2020. (**A**) Monthly average electricity generation by all sources. (**B**) Monthly average electricity generation by renewables.

Currently, seasonal fluctuations in electricity demand are largely handled by coal and gas (Fig. 5A). If the additional electricity demand from building electrification is met with electricity generation resembling the current grid, combustion emissions will shift from buildings to power plants. This can be avoided by generating this electricity from renewables. To provide some illustrative scenarios of how electricity generation could be met with renewables, we model scenarios where this demand is met by scaling up either wind or solar energy, using the existing monthly generation profiles (Fig. 5B).

**Figure 6:**
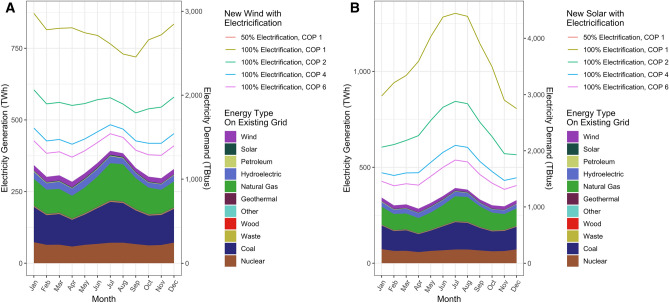
Additional renewable electricity needed to meet building electricity demand under different building electrification scenarios. (**A**) Shaded areas represent current (March 2010–February 2020) electricity generation, and lines represent the additional wind generation necessary to meet new demand under different building electrification scenarios if this additional demand were to be met by wind. (**B**) Shaded areas represent current (March 2010–February 2020) electricity generation, and lines represent the additional solar PV generation necessary to meet new demand under different building electrification scenarios if this additional demand were to be met by solar PV.

Meeting the 534 TWh gap in January electricity demand that would result with electrification using technologies with COP of 1 with wind would require scaling up wind from the average of ~ 19 TWh generation in January by a factor of ~ 28× (Fig. 6A). With a COP of 6, this demand could be met by increasing current wind generation by a factor of ~ 4.5× (Fig. 6A). In both scenarios, this would result in electricity generation exceeding supply in some months. With technologies with a COP of 1, grid generation would exceed demand by, at its highest, roughly 1.8× in April, when demand is low and wind generation is high. With COP of 6, grid generation exceeds demand by only 20% (Fig. 6A). If this is met by solar, with technology with COP 1, January solar generation would have to increase by a factor of ~ 303× (Fig. 6B). With technology with a COP of 6, January solar generation would only have to increase by a factor of ~ 36 × to meet January demand (Fig. 6B). With COP of 1, generation exceeds demand by a factor of 2.9 × in June (Fig. 6B); with COP of 6, June generation exceeds demand by ~ 40% (Fig. 6B). In all scenarios, the amount of overgeneration in off-peak months and the need for renewable energy deployment could be reduced by deployment of seasonal-scale electricity storage technologies.

## Discussion

We found a strong seasonal fluctuation in total energy consumption in the U.S., largely driven by winter heating demand for buildings. This “falcon curve” is not represented in many of the existing decarbonization pathways^1,2,5–8^. Currently, this fluctuation is managed largely by the existing fleet of UGS and LNG facilities, and other storage capacity intrinsic to existing fossil fuel energy systems. The existing UGS and LNG facilities, along with in-home and midstream storage capacity for wood, propane, fuel oil, and other home heating fuels represents a massive and crucial long-term energy storage resource that is essential to the current management of seasonal fluctuations in building energy demand in the U.S. Our results show that electrifying building heating will superimpose the seasonal demand fluctuation of the falcon curve onto the electrical grid. This will likely increase winter electricity demand enough to switch electricity generation from summer peaking to winter peaking, a phenomenon which has been shown in other studies that incorporated seasonality of energy demand^6,17,23–27^. With the current grid, this demand would likely be met by dispatchable electricity from gas and coal, which has long-term storage available. Since peak renewable energy production, especially for solar, does not coincide with peak heating demand, meeting this demand with renewables alone will require massive deployment of renewables on top of existing fossil generation^7,11,27^.

Our analysis uses historical energy use data, so potential changes in future building heating demand from factors including climate change, migration, building stock changes, and other changes in building energy consumption are not included. Future energy policies, new technologies for generation or storage, and electrification of other sectors may affect these dynamics in the future. We also assume that all current use of fossil fuels in buildings could be converted to electricity. The COPs used here are prototypical and intended for benchmarking—they do not reflect changes in COPs due to diurnal, seasonal, and spatial variations of outdoor temperatures, especially relevant for air source heat pumps (ASHP) during winter^17–19,22,30^. That said, our analyses of the historical energy system performance reveal the extent of the seasonal fluctuations in current total building energy demand and the role that fuel storage, especially from UGS, has in managing the falcon curve currently. Successfully electrifying buildings will require replacing or bypassing this existing storage capacity.

There are a number of strategies that can be used to manage the falcon curve. High COP heating and cooling technologies—such as ASHPs, ground source heat pumps (GSHPs), and networked GSHPs—can flatten the falcon curve on the building demand side by reducing the winter peak in electricity demand, therefore reducing demand placed on the electrical grid. Passive and active building energy efficiency, peak-shaving, and energy storage in buildings can support this as well, by either decreasing energy consumption or moving energy demand in time. Dispatchable renewable energy and large-scale deployment of long-term or seasonal electricity storage of capacity similar to existing UGS facilities may also be viable strategies for managing the demand placed on the electrical grid. Long-term electricity storage can also have a role in managing seasonal fluctuations in energy demand—helping to “flatten” the falcon curve as it is superimposed on the electrical grid. Long-term electricity storage would allow excess electricity generated by renewables in summer months to be stored and used for heating in winter months^8,28,29^, potentially reducing the increased deployment of renewable electricity necessary to meet this new demand with renewable electricity. However, storage capacity of this scale would require an expansion of the current design space, and may require advancements in chemistry, physics, or materials to develop technology capable of meeting this demand^29,31^.

To avoid unintended adverse consequences for climate, health, and environmental justice, building electrification and grid electricity need to be planned in tandem. For building electrification to maximize reductions in GHG and air pollutant emissions and make the most progress toward environmental justice, induced electricity demand should be met with non-combustion renewables. Conversion of current on-site fossil fuel use to combustion of renewable natural gas, hydrogen, biomass, or other renewable fuels either on-site or for electricity generation, may perpetuate the air pollution and health burden of building energy use, even if these fuels are truly GHG neutral^4,32,33^. Since seasonal differences in air pollution emissions have different health impacts, there is a role for atmospheric and public health scientists in this research^34^. Future decarbonization pathway development should incorporate seasonal fluctuations in building energy demand, and model scenarios for buildings and the electrical grid in tandem, in order to ensure that the electrical grid is capable of meeting building demand for space heating. Additionally, deployment, field testing, and further development of high-COP building heating and cooling technologies now can begin to flatten the falcon now, putting the building energy system on a trajectory well-aligned toward a zero-emissions future. In order to ensure that decarbonization makes the most progress possible toward correcting existing public health burdens and environmental injustices, and not producing new environmental injustices or impacts to public health, future work should include public health and atmospheric scientists in energy planning, alongside physicists, economists, energy modelers, and climate scientists.

Our research points toward several areas for future research. Future work should formally model different scenarios of long-term energy storage deployment to test the ability of long-term storage to alleviate the need for increased renewable energy deployment to meet the demand from electrified buildings. This may point toward future research in chemistry, physics, engineering, and/or materials science to develop new long-term energy storage technologies. Within the U.S., there is also likely to be variation in the seasonal fluctuation in building energy demand, which was not incorporated here. Evaluating the falcon curve for different regions or states may reveal fundamentally different dynamics in different regions, which then lead to different strategies to manage the falcon curve. Additionally, since this is relevant to mitigating both climate change and air pollution, future work could evaluate the magnitude and distribution of air quality and health consequences of different building decarbonization strategies.

## Conclusions

Here, we find strong seasonal fluctuation in total building energy demand, currently being managed by fossil fuels with long-term storage capacity. Further, we find that if buildings are decarbonized using inefficient electrification technologies, this will dramatically increase demand for electricity, especially in winter, producing the “falcon curve”. Even under high-efficiency building electrification, the U.S. electrical grid will likely switch from peaking in summer to winter. This represents a fundamental change in seasonal dynamics of the grid. For building electrification to truly represent healthy decarbonization of building energy, the additional electricity demand needs to be met with non-combustion renewable energy, which under our most optimistic scenario will require increasing wind generation by 4.5×. Seasonal fluctuations in building energy demand are currently being met largely by a fossil energy system with long-term energy storage. Development and deployment of long-term electricity storage may have a strong role in aiding renewable electricity in meeting the demand from newly electrified buildings.

## References

[1] International Energy Agency. Net Zero by 2050—A Roadmap for the Global Energy Sector. 224 (2021).

[2] Committee on Accelerating Decarbonization in the United States, Board on Energy and Environmental Systems, Division on Engineering and Physical Sciences, & National Academies of Sciences, Engineering, and Medicine. *Accelerating Decarbonization of the U.S. Energy System*. 25932 (National Academies Press, 2021). 10.17226/25932.

[3] Jenkins JD, Mayfield EN, Larson ED, Pacala SW, Greig C (2021). Mission net-zero America: The nation-building path to a prosperous, net-zero emissions economy. Joule.

[4] Buonocore JJ, Salimifard P, Michanowicz DR, Allen JG (2021). A decade of the US energy mix transitioning away from coal: historical reconstruction of the reductions in the public health burden of energy. Environ. Res. Lett..

[5] U.S. Energy Information Administration. Annual Energy Outlook 2021. https://www.eia.gov/outlooks/aeo/pdf/AEO_Narrative_2021.pdf (2021).

[6] White PR, Rhodes JD, Wilson EJH, Webber ME (2021). Quantifying the impact of residential space heating electrification on the Texas electric grid. Appl. Energy.

[7] Williams JH (2021). Carbon-neutral pathways for the United States. AGU Advances.

[8] Jenkins JD, Sepulveda NA (2021). Long-duration energy storage: A blueprint for research and innovation. Joule.

[9] Energy Information Administration (EIA). Commercial Buildings Energy Consumption Survey (CBECS) Data. https://www.eia.gov/consumption/commercial/data/2018/index.php?view=characteristics (2018).

[10] Energy Information Administration (EIA). Residential Energy Consumption Survey (RECS). https://www.eia.gov/consumption/residential/data/2015/ (2015).

[11] U.S. Energy Information Administration. Total Energy Monthly Data. https://www.eia.gov/totalenergy/data/monthly/index.php.

[12] Michanowicz DR (2017). A national assessment of underground natural gas storage: Identifying wells with designs likely vulnerable to a single-point-of-failure. Environ. Res. Lett..

[13] Mai, T. T. *et al. Electrification Futures Study: Scenarios of Electric Technology Adoption and Power Consumption for the United States*. NREL/TP--6A20–71500, 1459351 http://www.osti.gov/servlets/purl/1459351/ (2018). 10.2172/1459351.

[14] Natural Gas Storage Dashboard. https://www.eia.gov/naturalgas/storage/dashboard/.

[15] Nadel, S. *Comparative Energy Use of Residential Gas Furnaces and Electric Heat Pumps*. 29 https://www.aceee.org/sites/default/files/publications/researchreports/a1602.pdf (2016).

[16] Energy Star. Furnaces Key Product Criteria. *Furnances Key Product Criteria* https://www.energystar.gov/products/heating_cooling/furnaces/key_product_criteria.

[17] Jadun, P. *et al. Electrification Futures Study: End-Use Electric Technology Cost and Performance Projections through 2050*. 108 https://www.nrel.gov/docs/fy18osti/70485.pdf (2017).

[18] Liu, X., Anderson, A., Hughes, P. & Spitler, J. An Updated Assessment of the Technical Potential of Geothermal Heat Pump Applications in the United States. in *IGSHPA Technical/Research Conference and Expo* 9 (2017).

[19] Im, P. & Liu, X. Energy Performance Evaluation of a Recycled Water Heat Pump System in Cool and Dry Climate Zone. in *Proceedings of the IGSHPA Technical/Research Conference and Expo 2017* (International Ground Source Heat Pump Association, 2017). doi:10.22488/okstate.17.000524.

[20] U.S. Energy Information Administration. Natural Gas Annual Respondent Query System (EIA 191). https://www.eia.gov/naturalgas/ngqs/#?report=RP7&year1=2019&year2=2019&company=Name.

[21] Battery Storage in the United States: An Update on Market Trends. 33.

[22] Buffa S, Cozzini M, D’Antoni M, Baratieri M, Fedrizzi R (2019). 5th generation district heating and cooling systems: A review of existing cases in Europe. Renew. Sustain. Energy Rev..

[23] Bistline JET, Roney CW, McCollum DL, Blanford GJ (2021). Deep decarbonization impacts on electric load shapes and peak demand. Environ. Res. Lett..

[24] Bistline JET (2021). The importance of temporal resolution in modeling deep decarbonization of the electric power sector. Environ. Res. Lett..

[25] Ardani, K. *et al. Solar Futures Study*. 310 (2021).

[26] Wei M (2013). Deep carbon reductions in California require electrification and integration across economic sectors. Environ. Res. Lett..

[27] Tarroja B (2018). Translating climate change and heating system electrification impacts on building energy use to future greenhouse gas emissions and electric grid capacity requirements in California. Appl. Energy.

[28] Goetzler, W., Guernsey, M. & Kar, R. *Research and Development Roadmap. Geothermal (Ground-Source) Heat Pumps*. DOE/EE--0810, 1219848 http://www.osti.gov/servlets/purl/1219848/ (2012). 10.2172/1219848.

[29] Jenkins KEH (2021). The methodologies, geographies, and technologies of energy justice: A systematic and comprehensive review. Environ. Res. Lett..

[30] Sepulveda NA, Jenkins JD, Edington A, Mallapragada DS, Lester RK (2021). The design space for long-duration energy storage in decarbonized power systems. Nat. Energy.

[31] Sepulveda NA, Jenkins JD, de Sisternes FJ, Lester RK (2018). The Role of firm low-carbon electricity resources in deep decarbonization of power generation. Joule.

[32] Dedoussi IC, Eastham SD, Monier E, Barrett SRH (2020). Premature mortality related to United States cross-state air pollution. Nature.

[33] Lewis A (2021). Pollution from hydrogen fuel could widen inequality. Nature.

[34] Gilmore EA (2019). An inter-comparison of the social costs of air quality from reduced-complexity models. Environ. Res. Lett..

